# Revalorization of the Cooking Water (Aquafaba) from Soybean Varieties Generated as a By-Product of Food Manufacturing in Korea

**DOI:** 10.3390/foods10102287

**Published:** 2021-09-27

**Authors:** Esteban Echeverria-Jaramillo, Yoon-ha Kim, Ye-rim Nam, Yi-fan Zheng, Jae Youl Cho, Wan Soo Hong, Sang Jin Kang, Ji Hye Kim, Youn Young Shim, Weon-Sun Shin

**Affiliations:** 1 Department of Food and Nutrition, College of Human Ecology, Hanyang University, 17 Haengdang-dong, Seongdong-gu, Seoul 04763, Korea; estebangej@hanyang.ac.kr (E.E.-J.); yungha2@naver.com (Y.-h.K.); jeh6848@naver.com (Y.-r.N.); zyf054326@hanyang.ac.kr (Y.-f.Z.); 2 Department of Integrative Biotechnology, Sungkyunkwan University, 2066 Seobu-ro, Suwon 16419, Korea; jaecho@skku.edu; 3 Department of Foodservice Management and Nutrition, Sangmyung University, Seoul 51767, Korea; wshong@smu.ac.kr; 4 A-Life Corp, Goyang 10583, Korea; sangjin321@a-life.co.kr; 5 Department of Integrative Biotechnology, Biomedical Institute for Convergence at SKKU, Sungkyunkwan University, Suwon 16419, Korea; kjhkjhmlml@skku.edu; 6 Department of Plant Sciences, University of Saskatchewan, Saskatoon, SK S7N 5A8, Canada; younyoung.shim@usask.ca

**Keywords:** food by-product, aquafaba, revalorization, sustainability, legume, egg-replacement, foam, emulsion, vegan

## Abstract

Concerns regarding sustainability have prompted the search of value in the by-products of food manufacturing. Such is the case of the cooking water (CW) of chickpeas, which has shown its potential as a vegan egg white replacement. This study aimed to characterize and compare the CW from three novel legumes (black soybeans, BSB; yellow soybeans, YSB; and small black beans, SBB) obtained from the processing of Korean soybean foods, and the widely used CW from chickpeas (CH), with regard to total polyphenol, total carbohydrate, and protein contents, and further compare their foaming and emulsifying abilities and stabilities. Compositional analysis revealed that all the studied legumes possessed higher values than CH for all parameters. Furthermore, the CW from these legumes exhibited enhanced functional properties, particularly foaming capacity and stability. Taken together, our results suggest that the CW from BSB, YSB, and SBB, sourced from the manufacturing of legume food products, has the potential of being revalorized as a plant-based functional ingredient for vegan product development.

## 1. Introduction

Humanity is constantly challenged to become more sustainable by reducing the impact infringed on the planet. Currently, food production is responsible for a remarkable environmental impact, being accountable for the generation of greenhouse gases, deforestation, reduction of biodiversity, water pollution, and lessening of non-ice or dessert land [1]. As a consequence, actions to reduce its influence to the detriment of the planet are imperative. The recovery of valuable components from the waste of the food processing industry is proposed as a strategy to achieve this goal [2].

Recently, the use of the water separated from cooked or canned chickpeas has been studied, identifying that it possesses the same functional properties as those of egg whites for preparing meringues and sauces such as mayonnaises [3,4], thus constituting a suitable option for vegan versions of such foods. This material has been named aquafaba, which is a compound word formed by *aqua* and *faba* (Latin for water and beans, respectively) [5]. Chickpeas (CH) (*Cicer arietinum* L.) are an annual legume of the family *Fabaceae*, typically cultivated in India and the Middle East [6]. They are rich in proteins, carbohydrates (primarily starch and fibers), minerals, and vitamins [7]. Similar to CH, but native to East Asia, are black soybeans (BSB) (*Glycine max* [L] *Merr.*), yellow soybeans (YSB) (*Glycine max* [L] *Merr.*), and small black beans (SBB) (*Rhynchosia nulubilis*).These legumes have been staples of traditional oriental medicines, as they have multiple potential therapeutic effects on cancer, diabetes, cardiovascular, and cerebral and neurodegenerative diseases, [8] in addition to promoting melanin synthesis [9], increasing calcium absorption, and producing overall bone health benefits for postmenopausal women [10].

Furthermore, soybeans are the main ingredient of various foodstuffs such as fermented pastes, soy sauce, tofu, and soymilk. In South Korea alone, the total domestic consumption in the market year 2021/22 was forecast to be 1.39 million metric tons, of which 340,000 would correspond to domestic food manufacturing [11], with a market size of processed foods based on soybeans that would exceeded approximately 865 million USD in 2010 [12]. Data on the amount of water discarded from the cooking of these legumes is not available; nevertheless, it can be inferred from the previous information that the wastewater generated from producers nationwide is significant, thus generating treatment costs and deterioration to the environment if not properly disposed of [12].

In recent years, plant-based proteins have received increasing attention as substitutes for animal-based proteins [13]. According to the International Vegetarian Union [14], the demand for healthy and sustainable plant-based foods is increasing based on the growing vegan population worldwide. Additionally, legumes are suitable crops for further exploitation due to their low allergenicity, relative low prices, sustainable production, high yields, and balanced nutrient profile [15]. The inclusion of plant-based proteins in the diet, in conjunction with the partial replacement of animal-based protein sources, has shown beneficial effects on the health of individuals. These benefits include a decrease in the risk of type 2 diabetes, metabolic syndrome, obesity, cardiovascular illness, and hypertension, as well as a reduction in the incidence of cancer [16,17]. As a consequence, a lower mortality has been associated with an increased ingestion of plant proteins over animal based ones, particularly when the latter are sourced from processed and red meats in individuals with a minimum of one lifestyle risk factor [18]. It has been suggested that the whole source of the protein is what determines these effects, since animal protein foods are commonly accompanied by high levels of saturated fat, cholesterol, and salt, while plant proteins often include fiber, unsaturated fats, and phytochemicals [17].

With the exception of CH, the composition of the cooking water (CW) derived from the aforementioned legumes and their functional characteristics, including foaming and emulsification, have not been thoroughly analyzed to this date. These functional properties are crucial factors in the manufacturing of vegan food products such as meringues, mayonnaise, and bakery foods. Therefore, the goal of this study was to revalorize a by-product of the food processing industry in Korea by investigating the characteristics of the CW derived from three novel legumes (BSB, YSB, and SBB). Moreover, since CH has been identified as a suitable egg-white replacer [3,4,19,20,21,22,23], a comparison with the aquafaba derived from it was established to explore the possibility of constituting a new source for a vegan plant-based food additive.

## 2. Materials and Methods

### 2.1. Materials

The CW from BSB, YSB, and SBB was collected from the production of fermented soybean paste from Hwawangsang Foods (Gyeongsangnam-do, Korea). All dry legumes were pressure cooked at a 2:3 legume to water ratio (*w*/*w*) without prior soaking, using the following procedure. Dry seeds were cooked in a traditional custom-made brick steamer with a pressure lid fitted on top and a gas supply on the bottom for a total of 160 min. Cooking began with 80 min of boiling with an open pressure valve; then, the valve was locked for 25 min until 101 kPa of added pressure was reached. Subsequently, the gas supply was turned off, and after 5 min, the valve was unlocked to start the release of pressure. After 50 min, with the legumes locked inside the pot, the lid was unlocked and the cooked legumes were drained. CW was collected and stored under refrigeration until being shipped for analysis. CH CW was drained from canned CH (Divella, Bari, Italy). The pH of each sample was measured with a benchtop pH meter (Hanna Instruments HI5221-01, Woonsocket, RI, USA) before further analyses. Protein and total carbohydrate kits were purchased from Sigma-Aldrich (St. Louis, MO, USA). All other reagents were of analytical grade.

### 2.2. Compositional Analysis

#### 2.2.1. Total Polyphenol Content (TPC)

The total phenolic content (TPC) of the CW was measured with a colorimetric assay using the method of Singleton and Rossi [24], with some modifications. Briefly, 0.2 N Folin-Ciocalteu reagent (2.5 mL) was added to a 15 mL falcon tube containing the sample (0.5 mL). After mixing, a 7.5% sodium carbonate solution (2 mL) was added, and the mixture was incubated in the dark for 2 h at 25 °C. After incubation, absorbance of the mixture was measured at 760 nm, using a microplate reader (Synergy HT Multi-microplate, Bio-Tek Instruments, Winooski, VT, USA). Gallic acid was used as the reference to construct a standard curve, and the results have been reported as grams of gallic acid equivalent (GAE). All measurements were conducted in triplicate.

#### 2.2.2. Total Carbohydrate Content

The total carbohydrate content was measured using the phenol-sulfuric acid method. First, the sample (0.5 mL) was dissolved in deionized water (0.5 mL) and centrifuged at 130,000× *g* for 5 min. An aliquot (0.5 mL) of the resulting supernatant was diluted to 1:15 (v/v) with water in a glass test tube, and 5 mL of sulfuric acid was added. The samples were shaken in a water bath and heated in a dry oven at 90 °C for 15 min. Subsequently, a 5% phenol solution (1 mL) was added, and the absorbance was measured at 490 nm, using a microplate reader (Synergy HT Multi-microplate, Bio-Tek Instruments, Winooski, VT, USA). Glucose was used as the reference to construct the standard curve. All measurements were conducted in triplicate.

#### 2.2.3. Protein Content

Protein concentration was measured using the bicinchoninic acid assay (BCA, Thermo Scientific, Waltham, MA, USA). Briefly, the sample (25 µL) and the BCA reagent (200 µL) were added to a 96-well plate. After shaking for 30 s, the plate was covered and incubated at 37 °C for 30 min, followed by cooling to 20 °C. Subsequently, the absorbance was measured at 562 nm, using a microplate reader (Synergy HT Multi-microplate, Bio-Tek Instruments, Winooski, VT, USA). Bovine serum albumin (BSA) was used as the reference protein to obtain a standard curve. All measurements were conducted in triplicate.

#### 2.2.4. SDS-PAGE

Sodium dodecyl sulfate-polyacrylamide gel electrophoresis (SDS-PAGE) was performed according to the method of Laemmli et al. [25]. Samples were dissolved in deionized water and then heated to 95 °C for 5 min with the sample buffer (0.5 M Tris-HCl, pH 6.8, 10% SDS, 50% glycerol, 1.0% bromophenol blue, and 99% β-mercaptoethanol). Subsequently, an aliquot (15 µL) of the sample solution was loaded into each well of the gel. Electrophoresis was performed at a constant voltage of 200 V in a vertical slab gel with a 15% acrylamide concentration. Following electrophoresis, the gel was stained with Coomassie brilliant blue for 30 min and then destained for 5 h.

#### 2.2.5. Foaming Capacity (FC) and Foam Stability (FS)

The FC and FS were evaluated using an Ultra-Turrax T25 homogenizer (IKA, Staufen, Germany). The foam was generated by homogenizing the samples (20 mL) at 14,000 rpm for 1 min. A graduated falcon was used to measure volume of the generated foam. All measurements were performed in triplicate.

FC and FS were calculated using the following equations of Hammershøj [20] et al. (2004):

(1) $$\mathrm{FC}\;\left(\%\right)=\frac{Vfoam,\;t=1-Vliquid\;}{Vliquid}\times 100$$

(2) $$\mathrm{FS}\;\left(\%\right)=\frac{Vfoam,\;t}{Vfoam,\;t=1}\times 100$$

where *Vfoam, t* corresponds to foam volume at time *t* = 1, 10, 30, 60, and 90 min, and $V_{liquid}$ corresponds to the initial liquid volume.

#### 2.2.6. Emulsifying Capacity (EC) and Emulsion Stability (ES)

The EC and ES were determined according to the method of García-Vaquero et al. [26], with slight modifications. Briefly, to prepare the emulsion, canola oil (6 mL) was added to the sample solution (4 mL) in two parts during homogenization at 14,000 rpm for 2 min using an Ultra-Turrax T25 homogenizer (IKA, Staufen, Germany). Subsequently, the emulsions were centrifuged at 3000× *g* for 15 min. The volume of the emulsion layer was then measured. EC was calculated as follows:

(3) $$\mathrm{EC}\;\left(\%\right)=\frac{V_{E}}{V_{T}}\times 100$$

where *V_E_* corresponds to the volume of the emulsion layer after centrifugation and *V_T_* is the total volume.

For determining ES, the emulsions were heated to 85 °C for 10 min and then cooled to ambient temperature. The volumes of the emulsions after heating were measured after further centrifugation at 3000× *g* for 10 min. ES was calculated using the following equation:

(4) $$\mathrm{ES}\;\left(\%\right)=\frac{V_{H}}{V_{i}}\times 100$$

where *V_H_* corresponds to the volume of the emulsion layer after heating and *Vi* is the volume of the original emulsion. All measurements were conducted in triplicate.

### 2.3. Statistical Analysis

Statistical analysis was performed using SPSS (version 24.0; SPSS Inc., Chicago, IL, USA). The results are presented as mean ± standard deviation (SD). One-way ANOVA was used to analyze the significant differences among treatments, and Duncan’s post-hoc test was used to assess statistical significance (*p* < 0.05).

## 3. Results and Discussion

### 3.1. Compositional Analysis

#### 3.1.1. Total Polyphenol Content (TPC) and pH

Table 1 displays the composition and pH of the CW of all the legumes. The pH was found to be slightly acidic with no difference among samples. Similar pH values were reported for the CW of haricot beans, chickpeas, green lentils, and yellow soybeans, which ranged from 6.07 to 6.47 [27]. The TPC of the CW from the studied legumes showed higher values for BSB and SBB than those of the CW from YSB and CH. This is in agreement with a previous study, which found a higher TPC in the hot water extracts of SBB than in that of YSB [28]. Furthermore, a study performed by Xu and Chang [29] determined that the decrease in the content of phenolic compounds after cooking was greater for black soybeans than for yellow soybeans, suggesting that the concentration of TPC in the CW of the dark variants was higher than that in the CW of yellow beans.

**Figure 1:**
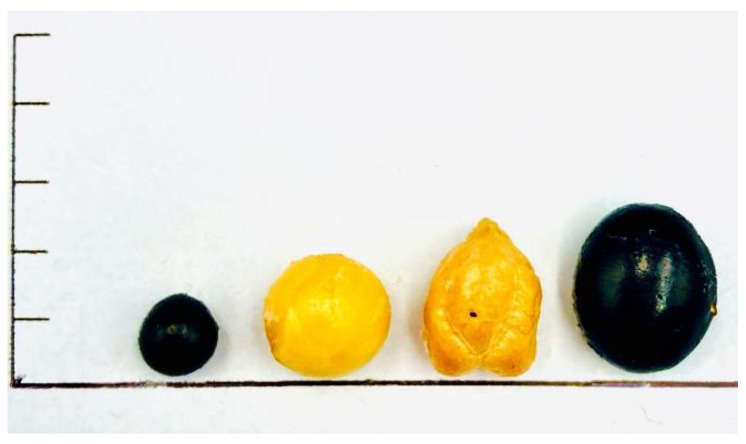
Images of the legumes tested in this study. From left to right: small black bean (SBB), yellow soybean (YSB), chickpea (CW), and black soybean (BSB). Horizontal lines across the vertical axis are equal to 0.5 cm.

Furthermore, the TPC among all the samples was significantly different (*p* < 0.05), with the CW of SBB presenting the highest amount. As displayed in Figure 1, SBB is comparatively smaller than the other three legumes, and consequently, it is likely that polyphenol compounds in the dry SBB migrated to the CW faster than in the case of the other legumes. These results are in agreement with those of Xu and Chang [30], who analyzed the CW of different legumes and observed that lentils, a small-sized seed, exhibited the highest TPC compared to larger legumes such as green peas, yellow peas, and chickpeas. Furthermore, Stantiall et al. [27] also suggested that the size of the seed influences the migration of compounds to the cooking water. Most phenolic compounds are found in the coat of the dark seeds [31,32,33]; therefore, the larger surface area of SBB allowed for a greater contact with the cooking fluid, thereby increasing the amount of water soluble compounds leaching into solution.

Our results suggest that regardless of the legume utilized, those examined in this study yielded CW with higher TPC than that for the CH CW, thus constituting a compositional advantage owing to the physiological activity of polyphenols. Moreover, the TPC values of 0.14 g GAE and 0.16 g GAE per 100 g of the CW of BSB and YSB, respectively, surpass those obtained by Damian, et al. [34] in the CW of whole green lentils, haricot beans, split yellow peas, and chickpeas, which ranged from 0.03 to 0.07 g GAE/100 g.

#### 3.1.2. Total Carbohydrates (CHO)

The data for CHO are provided in Table 1. The CW from all the three legumes possessed significantly higher CHO levels (*p* < 0.05) than that from CH, which had the lowest content. This was unexpected, as dry chickpeas contain 63.14 g CHO/100 g dry seed, a value that is more than double the amount of that of the other legumes, which have an average of 31.34 g CHO/100 g dry seed [35]. It has been reported that the type of carbohydrates that compose the CW of legumes such as chickpeas, haricot beans, green lentils, and split yellow peas corresponds primarily to water-soluble carbohydrates that are distinct according to their molecular weight. High-molecular weight carbohydrates consist of soluble fiber, while low-molecular weight carbohydrates correspond primarily to sucrose, raffinose, and stachyose [27]. Additionally, with regard to the CW of YSB, Serventi et al. [21] observed insoluble fiber derived from the seed coat. Therefore, it is likely that the carbohydrate profiles of the CW in the current study are similar to those reported previously, as the studied legumes in dry form contain soluble and insoluble fibers, oligosaccharides consisting of raffinose and stachyose, and sucrose [36,37].

Furthermore, the CHO obtained from the CW of the legumes hereby reported ranged from 4.74 to 5.48 g per 100 g CW (Table 1) and exceeded the values obtained by other authors, wherein the reported results varied from 1.82 to 4.12 for legumes such as haricot beans, yellow peas, lentils, and yellow soybeans [21,27,34]. However, it must be noted that the cooking conditions used in each of these studies differed from those used in the present research; unlike our study, none of them exceeded 90 min of cooking time. Thus, it is likely that the longer time of 160 min increased carbohydrate solubility from the seed to the cooking water. Nonetheless, this extended time was suitable, since it stimulated the greatest extraction of soluble solids while allowing the beans to remain intact and useful for the manufacturing of the intended products, as also reported by Carter [38].

#### 3.1.3. Protein Content

The total protein content is presented in Table 1. The data indicate that the protein content of the CW from the legumes in this study exceeded that of the CW from CH (*p* < 0.05). Additionally, the total protein of the CW from the legumes (BSB, 2.40 g; YSB, 1.51 g; SBB 3.19 g/100 g CW) was also higher than that obtained by other researchers, who reported values ranging from 0.95 to 1.50 g per 100 g of CH CW [4,27] and 0.68 g per 100 g of YSB CW [21]. This could be attributed to two factors. First, a longer cooking time was used in the present study compared to the 60- and 90-min cooking times used in the previous studies, which allowed a greater protein migration from the seed to the CW. Second, the macronutrient composition of the dry legumes prior to cooking differs among the legumes. SBB, BSB, and YSB have an average protein content of 37.40 g protein/100 g dry seed, which almost doubles that of chickpeas (17.27 g protein/100 g dry seed) [35]. It has been suggested that the concentration of substances in the CW is related to the loss of such substances in legumes after cooking [27]; hence, it was to be expected that more protein transferred to the water from the seeds that had a higher initial content.

Among the analyzed legumes, SBB had the highest protein content, which was likely due to the small size of this legume. According to Stantiall et al. (2018), protein solubilization during cooking is enhanced to a greater extent in smaller seeds than in larger varieties. Figure 1 contrasts each dry seed and exemplifies how SBB is distinguished from the other three due to its smaller size.

#### 3.1.4. SDS-PAGE Analysis

**Figure 2:**
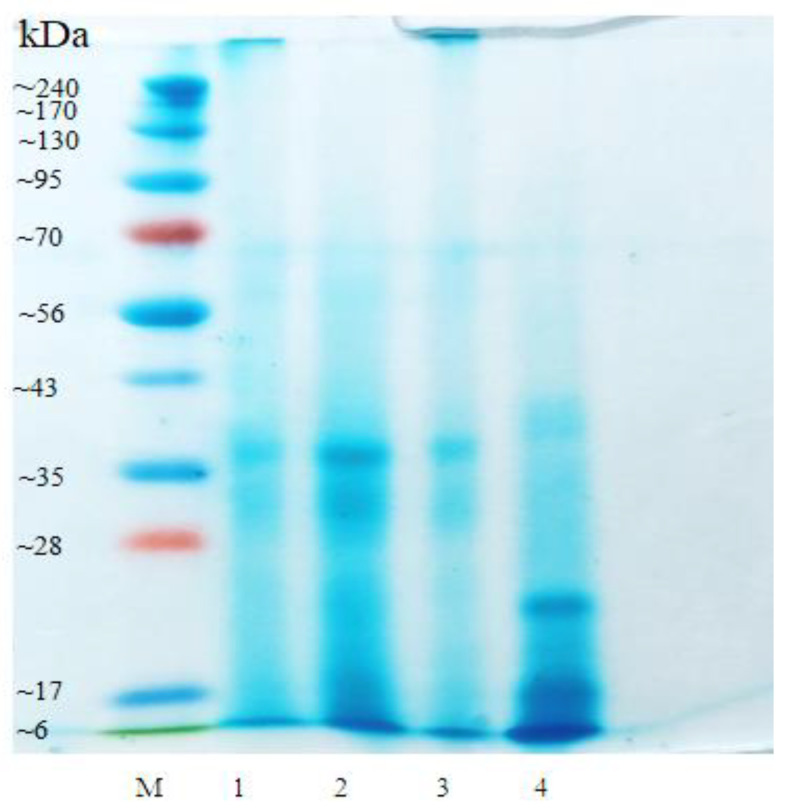
Protein profiles of the samples and the control. M: Protein marker, 1: BSB, 2: YSB, 3: SBB, 4: CH.

Figure 2 provides the protein profiles of the CW from BSB, YSB, SBB, and CH, showing bands of low-molecular weight proteins, ranging from ~6 to ~70 kDa, distributed similarly across all the samples. The protein profile of soybean seeds has been extensively described, with globulins and albumins being the two major storage protein fractions, followed by prolamins and glutelins in smaller amounts [39]. Among these, globulins are further subdivided into two major groups: glycinin (11S) and β-conglycinin (7S), with molecular weights of 150–200 and 300–380 kDa, respectively [40,41]. Nevertheless, only two publications have reported the characteristics of the proteins from the CW of a legume (chickpeas) [20,42], while no study has focused on the CW from soybeans. By comparison with a comprehensive profile of soybean proteins [40], it can be estimated that the proteins identified in the analyzed CW corresponds to 7S α (~70 kDa) and 7S β (~50 kDa) conglycinin, as well as acidic (~35 kDa) and basic (<15 kDa) 11S fractions of glycinin, in addition to albumin also possibly being present at the lowest range [4]. As mentioned, 11S and 7S globulins, in their native form, have higher molecular weights than those identified in the analyzed CW, indicating that the heat and pressure applied during cooking denatured their structure, thereby releasing smaller polypeptides into the solution. In partial agreement with this study, Buhl, Christensen, and Hammershøj [20] reported the protein profile of water from canned CH, only finding proteins below 24 kDa, which were identified as basic and acidic subunits of 11S legumin (23 kDa), γ-subunits of 7S vicilin (16 kDa), and subunits of 2S albumin (10 kDa and 12 kDa). Additionally, Raikos, Hayes, Agriopoulou, and Varzakas [42] carried out a proteomic analysis of canned chickpeas, finding a high variability in the profile of the samples, which presented nuclear, storage, and membrane proteins that solubilized from CH into the water.

### 3.2. Foaming Capacity (FC) and Foaming Stability (FS) Analysis

**Figure 3:**
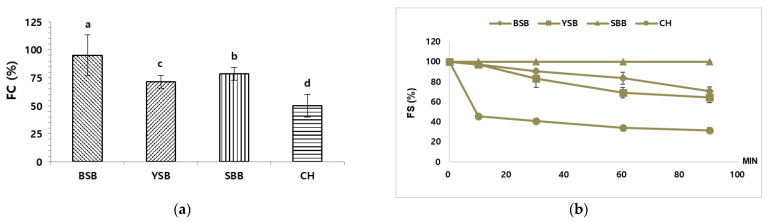
(**a**) Foaming capacity (FC) and (**b**) foaming stability (FS) of the CW from BSB, YSB, SBB, and CH. All values are expressed as mean ± standard deviation. (a–d) represent significant differences among samples (*p* < 0.05), according to Duncan’s test.

**Figure 4:**
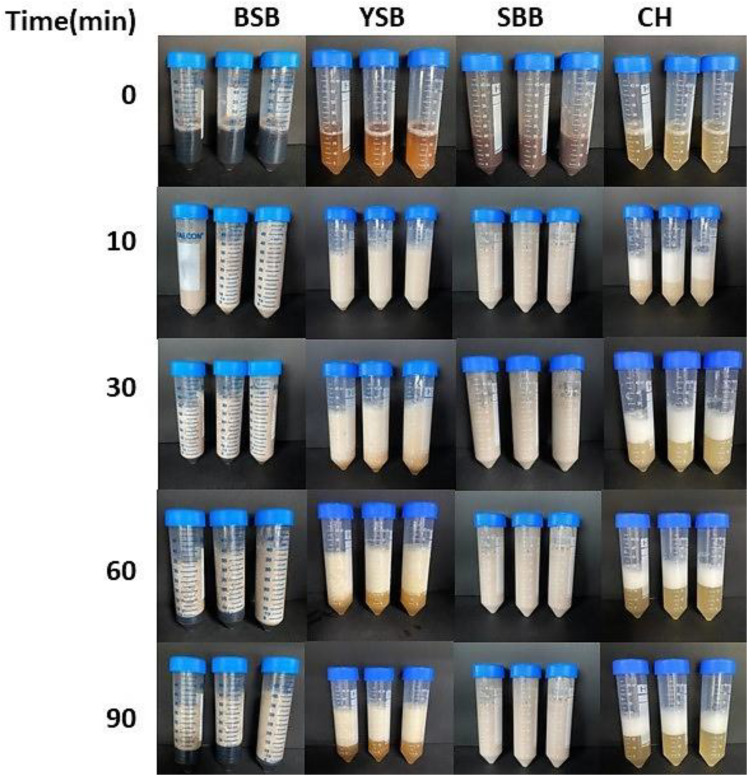
FS of the CW from BSB, YSB, SBB, and CH at 0, 10, 30, 60, and 90 min.

Figure 3 displays the values of FC and FS of the CW obtained from legumes and control (CH). FC of the CW from BSB was significantly higher than that of the CW prepared from the other types of legumes (*p* < 0.05), followed by the FC of the CW from SBB, YSB, and CH (Figure 3a). Additionally, with regard to FS, there were significant differences among all the CW, regardless of the source (Figure 3b). The CW from SBB showed the highest FS, followed by the CW from BSB, YSB, and CH. The stability of the foams of all types of CW, with the exception of that of SBB, decreased over 90 min, as can be seen in Figure 4. Furthermore, FS of the CW from CH declined rapidly during the 0–10 min interval. Based on these results, the CW from BSB exhibited the highest capacity to form a foam, while the most stable one corresponded to the CW from SBB.

It was expected that these results would be related to the protein and carbohydrate contents of the CW, since both macronutrients are of key importance for the formation and stabilization of foams [43]. They protect the interfacial film at the surface of the bubble against rupture and prevent or delay Oswalt ripening. Proteins are mainly involved in forming a layer at the air-water interface, relying on their hydrophobic groups, while carbohydrates, due to their hydrophilicity, tend to form complexes with the adsorbed proteins. This interaction increases viscosity and stabilizes the surface of the bubble, delaying liquid drainage [44]. Consequently, high protein levels, together with the presence of water soluble carbohydrates, result in good FC and high FS, due to conformational rearrangements and rapid adsorption at the air-water interface, prompting the formation of an elastic adsorbed layer [45]. Furthermore, the size of the proteins of the CW (Figure 2) also has a role in the foaming properties of this material. The presence of low-molecular weight proteins is a sign of a high degree of denaturation of larger structures, which are unfolded and lack strong bonding. As a consequence, hydrophobicity increases, which in turn enhances foam stability [20]. To produce high-quality foamed products, it is necessary to generate and maintain the foam over time, making FC and FS essential functional characteristics [27,46]. Additionally, it is important to be able to generate a large amount of stable foam using the same amount of CW. The CW from BSB, YSB, and SBB were superior to that of CH in terms of FC and FS, thereby providing evidence that the CW from these legumes can be used in place of the CW from CH in food applications that require foaming.

### 3.3. Emulsion Capacity (EC) and Emulsion Stability (ES) Analysis

Table 2 summarizes the values obtained for EC and ES. The results revealed that the EC of the CW from BSB and SBB was higher than that of CW from YSB and CH. With regard to ES, there were no statistically significant differences (*p* < 0.05) among all the legumes, although ES was slightly higher for the CW from BSB. These results can potentially be correlated with the higher protein and total carbohydrate contents of BSB and SBB than those of YSB and CH. For the formation of an emulsion, proteins are necessary to reduce the interfacial tension between the water and oil phases [47] that occurs due to the presence of hydrophobic and hydrophilic groups, which have been reported in the CW of CH [48] Additionally, the solubilized carbohydrates present in the CW are expected to possess interfacial activity that enhances and stabilizes an emulsion in two ways. The first is by altering the viscosity of the aqueous phase and thereby slowing the movement of droplets; the second is by preventing flocculation and coalescence through adsorption at the surface of the oil droplet [49]. Furthermore, an interaction between carbohydrates and proteins is expected due to the formation of polysaccharide–protein complexes, as also observed in the CW from CH [50]. These complexes are predominantly negatively charged and stabilize repulsion forces at the surface of the droplets, which consequently increases the ES by decreasing possible flocculation or creaming [48]. Moreover, owing to the intense pressure and temperature applied during the cooking of legumes, a more disordered polysaccharide–protein complex could be formed due to the Maillard reaction. Such complexes have the potential to form a thick film that is adsorbed at the oil/water interface, thus improving EC and ES [51]. It is important to consider the polyphenol content of the CW from the legumes in this study, as it has been reported that these compounds can bind to proteins and polysaccharides, thereby altering their emulsification properties [48]. Moreover, it is not expected that there is a direct influence of the individual protein fractions on their own on the EC and ESI of the CW analyzed, due to the degree of denaturation evidenced by the presence of only low-molecular weight proteins (Figure 2). β-conglycinin (7S) has been reported to be responsible for better emulsifying properties than glycinin (11S) [41,52]; however, only in their native, not denatured form.

Additionally, the emulsification properties of the CW examined, with regard to EC, surpassed those of the CW from other legumes, such as green lentils, haricot beans, split yellow peas, chickpeas, and yellow soybeans, which showed EC values of less than 55% [27,34] and ES values equal to those of different cultivars of chickpea, ranging from 71.9 to 77.1% [48]. This suggests that the CW from the legumes of this study has the potential to readily form a stable emulsion compared to the CW from more common legumes, thereby rendering them suitable for use as raw materials for the development of emulsion-based food products.

## 4. Conclusions

This study characterized the wastewater generated from the manufacturing of soybean foods, aiming to revalorize this industry by-product, in addition to comparing this CW to the well stablished CH aquafaba as a vegan egg white replacer. It was demonstrated that the CW from BSB, YSB, and SBB possessed higher total polyphenol, total carbohydrate, and protein contents and exhibited improved foaming and emulsion properties compared to those observed in the CW from CH. Additionally, the CW from SBB was superior to that from the other tested legumes, particularly with regard to protein content and foaming stability. Based on these results, it is feasible that the CW from these legumes may replace CH aquafaba and egg white in the production of vegan foods, thereby constituting an alternative and valuable use of this derivative material. Nevertheless, this research was limited by the lack of a direct comparison with the functional properties of egg white; therefore, it is suggested that subsequent studies be performed to address this situation. Additionally, as the storage and transportation of liquids are inconvenient for the industry, it is recommended that future research focuses on drying methods to improve the handling and shelf life of CW.

## Figures and Tables

**Table 1:** Total polyphenol, total carbohydrate, and total protein contents per 100 g, and pH of CW from black soybeans (BSB), yellow soybeans (YSB), small black beans (SBB), and chickpeas (CH). All values are expressed as mean ± standard deviation.

Composition (g/100 g CW)	Total Polyphenol	Total Carbohydrate	Protein	pH
BSB	0.14 ± 0.00 ^b^	5.48 ± 0.72 ^a^	2.40 ± 0.23 ^b^	6.26 ± 0.03 ^a^
YSB	0.07 ± 0.00 ^c^	4.74 ± 0.47 ^ab^	1.51 ± 0.04 ^c^	6.22 ± 0.03 ^a^
SBB	0.16 ± 0.00 ^a^	5.97 ± 1.3 ^a^	3.19 ± 0.18 ^a^	6.25 ± 0.02 ^a^
CH	0.03 ± 0.00 ^d^	3.28 ± 0.19 ^b^	0.39 ± 0.11 ^d^	6.23 ± 0.03 ^a^

^a–d^ represent significant differences among the samples (*p* < 0.05), according to Duncan’s test.

**Table 2:** Emulsifying capacity (EC) and emulsifying stability (ES) of the CW from BSB, YSB, SBB, and CH. All values are expressed as mean ± standard deviation.

Property (%)	BSB	YSB	SBB	CH
EC	80.76 ± 2.59 ^a^	75.48 ± 3.07 ^b^	78.89 ± 1.11 ^ab^	68.47 ± 1.68 ^c^
ES	76.85 ± 1.61 ^a^	72.58 ± 1.67 ^b^	75.11 ± 0.77 ^ab^	74.50 ± 1.80 ^ab^

^a–c^ represent significant differences among samples (*p* < 0.05), according to Duncan’s test.

## Data Availability

The data presented in this study are available in the article.
